# A Case of Polymicrobial Bacteremia in a Patient Undergoing Chemotherapy

**DOI:** 10.1155/2018/4165960

**Published:** 2018-01-15

**Authors:** Kyoko Arahata, Ryo Yamaguchi, Takeshi Terashima

**Affiliations:** ^1^Department of Internal Medicine, Tokyo Dental College Ichikawa General Hospital, 5-11-13 Sugano, Ichikawa, Chiba 272-0824, Japan; ^2^Department of Respiratory Medicine, Tokyo Dental College Ichikawa General Hospital, 5-11-13 Sugano, Ichikawa, Chiba 272-0824, Japan

## Abstract

We report an aggressive case of polymicrobial bacteremia in a patient with renal pelvis carcinoma. A 76-year-old man developed watery diarrhea after undergoing chemotherapy. He became unconscious and went into shock. Laboratory data showed severe neutropenia, renal failure, and lactic acidosis. Chest radiography showed multiple opacities. He died despite aggressive fluid resuscitation, catecholamine administration, antibiotic treatment, and mechanical ventilation. Blood culture isolates included *Escherichia coli*, *Klebsiella pneumoniae*, *Streptococcus pneumoniae*, *Acinetobacter baumannii*, and *Moraxella catarrhalis*. The foci of bacteremia were the respiratory tract and the bowel. The two infection foci and polymicrobial bacteremia are thought to be associated with the patient's poor prognosis. Although polymicrobial bacteremia is rare, awareness of this condition and of the rare causative pathogens, such as *A. baumannii* and *M. catarrhalis*, especially in patients with comorbidities and immunosuppression will help treat the patients with bacteremia.

## 1. Introduction

Sepsis and bacteremia are serious conditions that are associated with a high mortality. Comorbidities including diabetes mellitus, malignancies, chronic obstructive pulmonary disease (COPD), and hepatic cirrhosis, as well as immunosuppression following steroid therapy or antineoplastic therapy, are known risk factors for bacteremia [1]. The major sources of bacteremia are intravenous catheters, genitourinary, and respiratory tracts [2]. Among true bloodstream infection cases, only 6.2–9% have been reported to be polymicrobial [2–4].

Herein, we describe an aggressive case of polymicrobial bacteremia in a patient undergoing chemotherapy. The isolated microorganisms included the major pathogens *Escherichia coli*, *Klebsiella pneumoniae*, and *Streptococcus pneumoniae*, as well as the rare pathogens *Acinetobacter baumannii* and *Moraxella catarrhalis*.

## 2. Case Presentation

In April 2017, a 76-year-old man was transferred to our emergency room (ER) due to loss of consciousness. He had a medical history of COPD. Moreover, he had been diagnosed with left renal pelvis carcinoma in 2015 and was treated with 5 courses of gemcitabine and cisplatin (GC). The patient had undergone a left-sided nephroureterectomy in 2016.

Despite undergoing chemotherapy with GC following the surgery, multiple pulmonary metastases progressed. Second-line chemotherapy, consisting of methotrexate, vinblastine, adriamycin, and cisplatin, was administered, and pegylated granulocyte-colony stimulating factor was injected to prevent neutropenia on day 4. On day 7, the patient developed grade 2 watery diarrhea and was treated with loperamide. He was discharged on day 9. On day 10, watery diarrhea developed again, and the patient lost consciousness at home on day 11.

At the ER, the patient's Glasgow Coma Scale scores were 4 for E (eye opening), 1 for V (verbal response), and 2 for M (motor response). His condition was unstable. His blood pressure was not measurable. His pulse was palpable but very weak, with a pulse rate of 178 beats per minute (bpm). The patient's body temperature was 37.0°C, and the mucous membranes showed cyanosis. The patient's respiratory condition was unstable, with a respiratory rate of 21 breaths/minute. Oxygen supplementation therapy was started; the oxygen saturation was 92% at 10 L/min O_2_.

An initial laboratory examination showed a white blood cell count of 100/*µ*L with 18% neutrophils, platelet count of 3,000/*µ*L, hemoglobin level of 9.6 g/dL, C-reactive protein level of 32.09 mg/dL, blood urea nitrogen level of 45.6 mg/dL, creatinine level of 3.01 mg/dL, potassium level of 6.4 mEq/L, and glucose level of 36 mg/dL (Table 1). Blood gas analysis showed a pH of 7.29, PCO_2_ of 23.2 mmHg, PO_2_ of 64.5 mmHg, HCO_3_^−^ of 13.5 mmol/L, base excess of −14.4 mmol/L, and lactate level of 9.6 mmol/L, suggesting lactic acidosis. Chest radiography showed multiple opacities in both lungs, suggesting bacterial pneumonia and multiple pulmonary metastases.

As the patient did not respond to aggressive fluid resuscitation, pressors had to be administered. He also required intubation and mechanical ventilation. After admission, he was treated with cefepime and catecholamine at the intensive care unit (ICU). However, his blood pressure decreased gradually, and he died 4 hours after ER admission despite prompt antibiotic treatment. The patient's family declined an autopsy.

Before cefepime administration, two sets of blood cultures (one from the right femoral artery and one from the right medial cubital vein) were obtained sequentially via separate punctures. A total of 20 mL of blood were obtained for each set, and 10 mL were incubated in an aerobic and anaerobic bottle each. All blood cultures were processed by the hospital's microbiology laboratory, using the Bactec FX system (Becton Dickinson, NJ, USA). Antibiotic susceptibilities were assessed by a microliquid dilution method, according to the guidelines of the Clinical and Laboratory Standards Institute [5]. The blood cultures revealed the presence of *E. coli*, *K. pneumoniae*, and *S. pneumoniae* in the sample from the right femoral artery and *E. coli*, *S. pneumoniae*, *A. baumannii*, and *M. catarrhalis* in the sample from the right medial cubital vein (Table 2). Antibiotic susceptibility tests showed that *E. coli*, *K. pneumoniae*, and *A. baumannii* were susceptible to cefepime. The minimal inhibitory concentration of cefepime for these pathogens was <1 *μ*g/mL. Although no susceptibility test to cefepime was performed for *S. pneumoniae* and *M. catarrhalis*, *S. pneumoniae* was susceptible to penicillin G.

## 3. Discussion

In the present report, we describe an aggressive case of polymicrobial bacteremia. The most frequently isolated microorganisms causing true bacteremia are *Staphylococcus aureus*, *E. coli*, *Enterococcus* spp., *K. pneumoniae*, coagulase-negative staphylococci, *S. pneumoniae*, and *Pseudomonas aeruginosa*, and *A. baumannii* and *M. catarrhalis* rarely cause bacteremia [2–4, 6]. A study on bacteremia with febrile neutropenia showed that, among 50 analyzed episodes, a single pathogen was isolated in 35 cases, 2 pathogens in 10 cases, and ≥3 pathogens in the remaining 5 cases [7]. The present case is rare because 5 pathogens were isolated from the blood cultures.

It is critical to know whether the isolated bacteria were the cause of the bacteremia or only contaminants. *S. pneumoniae* and *E. coli* were isolated from 2 sets of blood cultures; both are known to be major causes of bacteremia. In a previous study, the proportions of clinically significant true bacteremia/contamination/unknown were reported to be 100%/0%/0% for *S. pneumoniae*, 97%/1%/2% for *E. coli*, 95%/1%/4% for *K. pneumoniae*, and 67%/0%/33% for *A. baumannii*. No data were obtained for *M. catarrhalis*. Isolated bacteria that are known to be typical contaminants include *Bacillus* spp., *Micrococcus* spp., *Corynebacterium* spp., and coagulase-negative staphylococci [2]. In the present case, we believe that *A. baumannii* and *M. catarrhalis* were not contaminants but rather they contributed to the clinical course. This consideration is substantiated by a report that showed that *A. baumannii* and *M. catarrhalis* are rarely isolated as contaminants [2].

Among the pathogens isolated from 50 episodes of febrile neutropenia, *A. baumannii* constituted 2.7% of the total isolates [7]. Although *A. baumannii* rarely causes bacteremia [2], it has also been shown that cases of *A. baumannii* bacteremia typically originate in the respiratory tract and that its risk factors include immunosuppression and COPD [1, 8]. As the patient in our case had COPD and pneumonia, the focus of infection was likely the respiratory tract. Alternatively, it could have been the abdomen as the second most common focus of infection for *A. baumannii* bacteremia has been shown to be the abdomen [1]. According to a previous analysis, of 95 patients with *A. baumannii* bacteremia, 50, 24, and 11 had respiratory tract, urinary tract, and intra-abdominal infections, respectively [9]. The patient in our case had watery diarrhea after undergoing chemotherapy. Severe neutropenia often causes bacterial translocation from the intrabowel space to the bloodstream.

To the best of our knowledge, documented cases of *M. catarrhalis* bacteremia are rare. Collazos et al. reported two cases of *M. catarrhalis* bacteremic pneumonia and a review of the literature [10]. In their review, 4 of the 9 patients had COPD as a comorbidity, and 2 patients were undergoing chemotherapy. In another study, the incidence of bacteremia in patients with COPD was 2.5 higher when compared to that of the general population, and 1 case of *M. catarrhalis* bacteremia was reported in the COPD group [11]. Another case of *M. catarrhalis* bacteremia caused by pulmonary infection was reported in an immunosuppressed patient [12]. Considering previous research, the focus of *M. catarrhalis* bacteremia in our case was likely the respiratory tract.

The neutropenia that was seen in our case suggests that the patient had an immunosuppressive condition after chemotherapy. Alternatively, neutropenia might have been caused by his extremely critical condition as the results of the physical examination, laboratory data, and the clinical course indicated that he was in septic shock [13]. It is likely that both factors (immunosuppression and septic shock) resulted in severe neutropenia and thrombocytopenia. The clinical picture of the present case was characterized by septic shock and multiple organ failure, which resulted in death at the ICU despite antibiotic administration, fluid infusion, catecholamine administration, and mechanical ventilation.

The antibiotic susceptibility tests showed that *E. coli*, *K. pneumoniae*, and *A. baumannii* were susceptible to cefepime. Cefepime is known to be effective against *S. pneumoniae* and *M. catarrhalis* [14]. We believe that the condition of the patient was so severe due to the septic shock and multiple organ failure that even the early administration of cefepime did not improve the clinical course.

The mortality rate among patients with *A. baumannii* bacteremia is 43.5–58% [1, 8, 9]. It is known that shock, extremely elevated white blood cell levels, the presence of a malignancy, and elevated serum creatinine levels are associated with poor prognosis [2, 6]. Besides these factors, two foci of infection (the respiratory tract and bowel) and polymicrobial bacteremia might have been associated with the death in our case [2, 6, 15].

Bacteremia is particularly common in patients with neutropenia because of a combination of epithelial damage due to chemotherapy and immunosuppression. Bacteremia is thought to occur via bacterial translocation across the mucosal barriers of the epithelium under chemotherapy, particularly by impaired colonization resistance and domination of pathogenic bacteria [16]. In our case, severe damage of the intestinal and bronchial epithelia might explain the bacteremia from two foci.

In summary, we describe an aggressive case of bacteremia in which 5 pathogens were isolated from blood cultures in an immunosuppressed patient after chemotherapy. The foci of bacteremia were likely the respiratory tract and the bowel. Although polymicrobial bacteremia is rare, awareness of this condition and of the rare causative pathogens, such as *A. baumannii* and *M. catarrhalis*, especially in patients with comorbidities and immunosuppression will help treat the patients with bacteremia.

## Figures and Tables

**Table 1:** Results of the laboratory examinations.

Hematology		Reference intervals	Chemistry		Reference intervals
Blood cell counts					
White blood cell (/*µ*L)	100	3,500–8,500	TP (g/dL)	4.9	6.7–8.3
Neutrophils (%)	18.2	40.0–70.0	Albumin (g/dL)	2.7	3.8–5.2
Eosinophils (%)	0	1.0–6.0	TB (mg/dL)	0.6	0.2–1.2
Lymphocytes (%)	81.8	20.0–50.0	AST (U/L)	66	10–40
RBC (×10^4^/*µ*L)	270	430–570	ALT (U/L)	40	5–45
Hb (g/dL)	9.6	13.5–17.0	LD (U/L)	257	115–250
Hct (%)	28.5	40.0–50.0	ALP (U/L)	197	115–380
Platelets (/*µ*L)	3,000	150,000–350,000	CK (U/L)	53	50–220
			Amylase (U/L)	54	40–130
Coagulation			TC (mg/dL)	105	130–219
PT (seconds)	14.5		TG (mg/dL)	194	30–149
PT (%)	77.6	70.0–140.0	BUN (mg/dL)	45.6	8.0–20.0
PT-INR	1.11	0.80–1.20	Cr (mg/dL)	3.01	0.61–1.04
APTT (seconds)	54.8	<40	UA (mg/dL)	9.0	3.8–7.0
Fibrinogen (mg/dL)	540	200–400	Na (mEq/L)	145	137–147
FDP (*µ*g/mL)	22.7	<10.0	K (mEq/L)	6.4	3.5–5.0
D-dimer (*µ*g/mL)	9.6	<1.0	Cl (mEq/L)	111	98–108
			Ca (mg/dL)	8.2	8.4–10.4
Blood gas analysis			P (mg/dL)	4.6	2.5–4.5
(O_2_ 10 L)			BS (mg/dL)	36	70–110
pH	7.29	7.350–7.450	CRP (mg/dL)	32.09	<0.30
PCO_2_ (mmHg)	23.2	35.0–45.0			
PO_2_ (mmHg)	64.5	75.0–100.0			
HCO_3_^−^ (mmol/L)	13.5	20.0–26.0			
BE (mmol/L)	−14.4	±3.0			
Lactate (mmol/L)	9.6	0.5–1.6			

**Table 2:** Blood culture results.

Isolated pathogen	Suspected focus of bacteremia
*Escherichia coli*	Bowel
*Klebsiella pneumoniae*	Bowel and respiratory tract
*Streptococcus pneumoniae*	Respiratory tract
*Acinetobacter baumannii*	Respiratory tract and bowel
*Moraxella catarrhalis*	Respiratory tract
